# Genome sequence resource for “*Candidatus* Liberibacter asiaticus” strain GDCZ from a historical HLB endemic region in China

**DOI:** 10.1186/s12863-023-01160-3

**Published:** 2023-11-04

**Authors:** Yongqin Zheng, Yun Li, Pengbin Xu, Chaoji Liu, Jianchi Chen, Xiaoling Deng, Zheng Zheng

**Affiliations:** 1 National Key Laboratory of Green Pesticide, South China Agricultural University, Guangzhou, Guangdong China; 2 Guangdong Province Key Laboratory of Microbial Signals and Disease Control, South China Agricultural University, Guangzhou, Guangdong China; 3 Chaozhou Fruit Research Institute, Chaozhou, Guangdong China; 4 San Joaquin Valley Agricultural Sciences Center, Agricultural Research Service, United States Department of Agriculture, Parlier, CA USA

**Keywords:** *Candidatus* Liberibacter asiaticus, Huanglongbing, Historical origin, PacBio long-read sequencing

## Abstract

**Objectives:**

“*Candidatus* Liberibacter asiaticus” (CLas) is an un-culturable α-proteobacterium that caused citrus Huanglongbing (HLB), a destructive disease threatening citrus production worldwide. In China, the presence of HLB was first reported in Chaoshan region of Guangdong province, China around a century ago. Thus, whole genome information of CLas strains from Chaoshan area become the most important resource to understand the population diversity and evaluation of CLas in China.

**Data description:**

CLas strain GDCZ was originally from Chaozhou city (Chaoshan area) and sequenced using PacBio Sequel long-read sequencing and Illumina short-read sequencing. The genome of strain GDCZ comprised of 1,230,507 bp with an average G + C content of 36.4%, along with a circular CLasMV1 phage: CLasMV1_GDCZ (8,869 bp). The CLas strain GDCZ contained a Type 2 prophage (37,452 bp) and encoded a total of 1,057 open reading frames and 53 RNA genes. The whole genome sequence of CLas strain GDCZ from the historical HLB endemic region in China will serve as a useful resource for further analyses of CLas evolution and HLB epidemiology in China and world.

## Objective

“*Candidatus* Liberibacter asiaticus” (CLas) is a fastidious phloem-limited Gram-negative bacterial pathogen causing citrus Huanglongbing (HLB, yellow shoot disease), the most destructive disease that threatening citrus production worldwide. Nearly all commercial cultivars were susceptible to HLB [1]. HLB was first observed in Chaoshan area, east of Guangdong Province, China, in late 1890s [2, 3]. Severe outbreaks of HLB were reported in the citrus growing area of Guangdong Province around 1940s [4]. Based on HLB spreading timeline, it is generally believed that from Chaozhou region, HLB spread to Pearl River Delta area in central Guangdong through the movement of infected nursery stocks [2]. Now, HLB is found in 11 provinces in southern China, causing significant economic loss in Chinese citrus production. CLas strains from Chaoshan area become the most important resource to understand the evaluation and population diversity of CLas in China. Therefore, genomic information of CLas strain from Chaoshan area are needed.

CLas is by far not culturable in vitro. Studies on CLas genome are mainly limited to analyses in planta or in Asian citrus psyllid (ACP, *Diaphorina citri*). Population diversity of CLas provided baseline information for research and HLB management in China and the world. Thanks for the advancement of next generation sequencing (NGS) technologies, CLas genome sequences can now be acquired from ACP [5] and infected plants [6]. A comprehensive collection of CLas genome sequences from different geographical and ecological locations is fundamental for CLas population analysis. There are currently 45 CLas genome sequences deposit in NCBI Genome database. All the CLas genome sequences, except strain JRPAMB1, was short-reads sequencing-based. The genome of strain JRPAMB1 originally from Florida was assembled from PacBio long-read sequencing. Both short-reads and long-reads sequencing formats have advantages and disadvantages. The research was set to take advantages of both sequencing formats to obtain a high-quality bacterial genome.

## Data description

CLas strain GDCZ was originally collected from a HLB-affected fruit of *Citrus reticulata* cv. *Tankan* showing HLB symptoms (small asymmetrical fruit with uneven coloring of fruit) in a citrus orchard (23°40″22″N, 116°38″33″E, 25 m) located in Chaozhou City (Chaoshan area), Guangdong Province, China. DNA was extracted from fruit piths because of the high concentration of CLas [7]. Total DNA was extracted from fruit piths using an E.Z.N.A. High-Performance Plant DNA Extraction Kit (Omega Bio-Tek Co., China). The presence of CLas was confirmed by a real-time quantitative PCR with primer set CLas4G / HLBr [8] with cycle threshold (Ct) value = 21.73. Phage typing PCR with phage specific primer sets [9, 10] showed that CLas strain GDCZ contained a Type 2 phage and a CLasMV1 phage.

Genome sequencing was performed by a PacBio Sequel system with 20-kb library insert size (Pacific Biosciences, Menlo Park, CA, U.S.A.) and an Illumina Hiseq Xten platform with 150-bp paired-end output (Illumina Inc., San Diego, CA, U.S.A.) through a commercial source. In total, 2,328,216 clean long-reads with a length range from 5,374 to 111,541 bp (N50 length = 6,426 bp) and 95,698,604 clean short-reads (150-bp) were generated from the GDCZ sample (Table 1, Data file 1) [11].

**Table 1:** Overview of data files/data sets

**Label**	**Name of data file/data set**	**File types (file extension)**	**Data repository and identifier (DOI or accession number)**
Data file 1	A workflow of genome assembly for CLas strain GDCZ	Spreadsheet Format file (.xlsx)	Figshare (https://doi.org/10.6084/m9.figshare.23614437.v2) [11]
Data file 2	Prophage detection of CLas strain GDCZ	Spreadsheet Format file (.xlsx)	Figshare (https://doi.org/10.6084/m9.figshare.23614437.v2) [11]
Data file 3	ANI values for CLas strain GDCZ	Spreadsheet Format file (.xlsx)	Figshare (https://doi.org/10.6084/m9.figshare.23614437.v2) [11]
Data file 4	Cluster analyses	Portable Document Format file (.pdf)	Figshare (https://doi.org/10.6084/m9.figshare.23614437.v2) [11]
Data set 1	Sequencing long-reads of CLas strain GDCZ	Fasta file (.fa)	NCBI SRA (https://identifiers.org/ncbi/insdc.sra:SRR23622213) [12]
Data set 2	Sequencing short-reads of CLas strain GDCZ	Fasta file (.fa)	NCBI SRA (https://identifiers.org/ncbi/insdc.sra:SRR23622214) [13]
Data set 3	Genome assembly of CLas strain GDCZ	Genbank file (.gb)	NCBI GenBank (https://identifiers.org/ncbi/insdc:CP118922) [14]

All reads mapped to *Citrus maxima* genome (MKYQ00000001.1), *C. reticulata* genome (NIHA00000000.1), *C. sinensis* genome (AJPS00000000.1), *C. sinensis* mitochondrion (NC_037463.1) and *C. reticulata* chloroplast (KU170678.1) were removed using Bowtie2 v2.4.1 (for short-reads) and BWA v0.7 (for long-reads) with default settings [15, 16]. A total of 152,762 (6.56%) unmapped long-reads and 15,179,368 (15.86%) unmapped short-reads were retained for assembly (Data file 1) [11]. The de novo assembly was performed by Canu v2.1.1 (for long-reads) (genomeSize = 1.2 M, corrected ErrorRate = 0.40) and CLC Genomic Workbench v20.0 (for short-reads) (minimum contig length = 500 bp) [17]. A total of 65 contigs (N50 = 13,580 bp) were generated from the long-reads and 39,316 contigs (N50 = 857 bp) from the short-reads (Data file 1) [11]. Contig blast against strain A4 genome (CP010804.2) by BLAST + v2.12.0 [18] identified a total of 85 CLas contigs (62 from long-reads and 23 from short-reads), generating a GDCZ scaffold sequence. This scaffold had three segments apart by two inverted 222-bp repeat gaps (Data file 1) [11]. The two 222-bp gaps can be satisfactorily filled by reads mapping with Illumina short-reads. The final sequence was polished by short-reads mapping (Length fraction = 0.95, Similarity fraction = 0.95). These efforts generated the GDCZ whole-genome sequence with a total of 1,230,507 bp (with average G + C content of 36.4%). Coverage levels analysis of reads mapping to three types of known prophage genomes (Type 1: SC1, HQ377372.1; Type 2: SC2, HQ377373.1; Type 3: P-JXGC-3, KY661963.1) showed that CLas strain GDCZ only harbored a Type 2 prophage (93.38%, from position 1,193,056 to 1,230,507 bp) (Data file 2) [11]. In addition, a circular contig generated from long-reads (8,869 bp) by Canu v2.1.1 was identical as the CLasMV1 phage genome (CP045566.1). Genome annotation revealed that CLas strain GDCZ contained 1,057 open reading frames and 53 RNA genes.

The average nucleotide identity (ANI) was further analyzed between the strain GDCZ genome and 10 CLas genomes originally from China using FastANI v1.33 (Fragment length = 1,000 bp) [19] (Data file 3) [11]. Three distinct branches were generated based on ANI matrix (Data file 4) [11]. Particularly, strain GDCZ was clustered with two strains from Guangdong province (strain A4 and PGD) but far apart from CLas strains from others provinces (Jiangxi province: strain JXGZ and JXGC, Yunnan province: strain YNJS and PYN; Guangxi province: strain gxpsy).

Please see Table 1 for links to Data files 1–4 and Data sets 1–3.

## Limitations

Due to the current inability to culture in vitro, the high ratio of citrus DNA as compared to CLas DNA in total DNA made the CLas genome sequencing more challenging. Therefore, the strategy of genome sequencing of CLas required a higher sequencing depth to obtain more CLas reads for improving the quality of CLas genome assembly. In this study, genome assembly of PacBio long-reads sequencing of CLas DNA samples extracted from plant host sources was insufficient to obtain a complete CLas genome, which suggested that the sequencing depth of one PacBio long-reads sequencing run could not enough to cover the whole CLas genome. Thus, an addition Illumina Hiseq Sequencing for CLas GDCZ samples was performed and added with PacBio long-reads to obtain the complete high-quality CLas GDCZ genome. Further research on CLas DNA enrichment, e.g. removing of citrus host DNA or enriching bacterial cells before DNA extraction, can be established to increase the ratio of CLas DNA in total DNA from citrus host, which in turn to increase the ratio of CLas reads in sequencing data obtained by PacBio platform and improve the quality of assembly of CLas genome.

## Data Availability

The data described in this Data note can be freely and openly accessed on Figshare (https://figshare.com) [11]. Data set 1–3 are available on the NCBI database. The clean reads have been submitted to the NCBI Sequence Read Archive under the accession number SRR23622213 and SRR23622214 (Data set 1–2) [12, 13]. The genome assembly was submitted to NCBI Genome Database and is available under the accession number CP118922.1 (Data set 3) [14].

## Abbreviations

CLas: *Candidatus* Liberibacter asiaticus
HLB: Huanglongbing
PacBio: Pacific Biosciences
ACP: Asian Citrus Psyllid
NGS: Next Generation Sequencing
Ct: Cycle Threshold
BWA: Burrow-Wheeler Aligner
BLAST: Basic Local Alignment Search Tool
ANI: Average Nucleotide Identity
